# Optimisation of Deep Learning Small-Object Detectors with Novel Explainable Verification

**DOI:** 10.3390/s22155596

**Published:** 2022-07-26

**Authors:** Elhassan Mohamed, Konstantinos Sirlantzis, Gareth Howells, Sanaul Hoque

**Affiliations:** 1 School of Engineering, University of Kent, Canterbury CT2 7NT, UK; s.hoque@kent.ac.uk; 2 School of Computing, University of Kent, Canterbury CT2 7NZ, UK; w.g.j.howells@kent.ac.uk

**Keywords:** convolutional neural network, explainable artificial intelligence, small object detection

## Abstract

In this paper, we present a novel methodology based on machine learning for identifying the most appropriate from a set of available state-of-the-art object detectors for a given application. Our particular interest is to develop a road map for identifying verifiably optimal selections, especially for challenging applications such as detecting small objects in a mixed-size object dataset. State-of-the-art object detection systems often find the localisation of small-size objects challenging since most are usually trained on large-size objects. These contain abundant information as they occupy a large number of pixels relative to the total image size. This fact is normally exploited by the model during training and inference processes. To dissect and understand this process, our approach systematically examines detectors’ performances using two very distinct deep convolutional networks. The first is the single-stage YOLO V3 and the second is the double-stage Faster R-CNN. Specifically, our proposed method explores and visually illustrates the impact of feature extraction layers, number of anchor boxes, data augmentation, etc., utilising ideas from the field of explainable Artificial Intelligence (XAI). Our results, for example, show that multi-head YOLO V3 detectors trained using augmented data produce better performance even with a fewer number of anchor boxes. Moreover, robustness regarding the detector’s ability to explain how a specific decision was reached is investigated using different explanation techniques. Finally, two new visualisation techniques are proposed, WS-Grad and Concat-Grad, for identifying explanation cues of different detectors. These are applied to specific object detection tasks to illustrate their reliability and transparency with respect to the decision process. It is shown that the proposed techniques can result in high resolution and comprehensive heatmaps of the image areas, significantly affecting detector decisions as compared to the state-of-the-art techniques tested.

## 1. Introduction

Machine learning in general and deep learning in specific has demonstrated efficiency in a wide range of applications, such as chemical process analysis [1], defect detections [2] and medical image analysis [3]. Moreover, state-of-the-art object detector systems based on deep learning and convolutional neural networks have shown significant performances in terms of accuracy and speed on standard datasets [4,5]. Such datasets mainly contain large objects that occupy a large area of an image. This helps the detector to exploit more pixels during the training and inference stages. Consequently, more information can be used in the training and prediction steps. The case becomes more challenging for small size objects as the information available to the system for training or inference is limited. Furthermore, small size objects may appear in groups which further complicate their detection. When it comes to small-size objects, the performance of state-of-the-art systems has not been systematically investigated.

Anchor boxes that are estimated from the training data are used as initial priors to enhance the predicted bounding boxes. Small anchor boxes are efficient with small objects but fail to capture large ones and vice versa. This limits the capabilities of state-of-the-art object detectors to detect small and large size objects simultaneously. Thus, redesigning the model’s architecture and anchor boxes may help to tackle this problem. However, increasing the number of anchor boxes to fit both object sizes can negatively impact the detector’s speed and accuracy. Speed-accuracy trade-off is another major challenge for object detectors [6]. Therefore, object detectors are considered application-oriented.

This paper investigates the impact of different object detection architectures on detector performance. State-of-the-art object detectors are tested on an application-specific dataset. The proposed dataset is challenging as its major components are small size objects. Nevertheless, it contains large and medium size objects as well, which introduces more challenges. Optimisation parameters, such as the minimum number of anchor boxes that can efficiently represent the proposed datasets, which base network to use, which feature extraction layer to use and the impact of different detector architectures on the accuracy, are investigated. Besides, the training strategies and data augmentation implications are sought to be discussed.

The contribution of this paper is twofold:

- A deep investigation of state-of-the-art object detectors is presented on a challenging dataset that has multi-size objects but the majority of these objects are small size. The investigation presents a road map for researchers and developers to choose the most appropriate detector for a given application.
- Novel techniques to visualise the contributing features to the detection decision to ensure the reliability of the produced detectors are proposed. The proposed techniques, besides state-of-the-art ones, are applied for the object detection task. Explainable Artificial Intelligence (XAI) techniques are usually used for classification tasks. However, applying these techniques for a different task (object detection) is a novel contribution.

This paper is organised as follows: state-of-the-art systems are presented in Section 2. The methodology is introduced in Section 3, where the system architecture, the dataset, the training parameters and the evaluation metrics are discussed. In Section 4, results are presented and discussed. Section 5 proposes novel visualisation techniques for convolutional neural networks predictions with application to object detection tasks. Finally, the paper is concluded in Section 6.

## 2. State-of-the-Art

Object detection architectures can be split into two main categories: one-stage and two-stage detectors. Unlike one-stage detectors, two-stage detectors have an extra step to generate region proposals. Every generation of the region proposal-based methods offer an improvement over the previous generation. For instance, Spatial Pyramid Pooling (SPP) network [7] modified region-based Convolutional Neural Network (R-CNN) [8] with an SPP layer. The SPP layer uses the convolutional layer’s feature map to generate fixed-size vectors for object proposals. Fast R-CNN [9] is proposed to overcome R-CNN and SPP-Net problems. Without caching features, it can be trained end-to-end on a multi-task loss function for classification and bounding box regression. Fast R-CNN uses a special case of SPP layer called the Region of Interest (RoI) pooling layer, which has one pyramid level. In addition, softmax classifier that is used in Fast R-CNN outperforms linear Support Vector Machines (SVMs) classifiers used in R-CNN and SPP-Net. Further, it does not require disk storage. Besides, it improves both accuracy and efficiency.

Object detection algorithms such as SSP-Net and Fast R-CNN suffer from bottleneck computations due to region proposals. Ren et al. [5] introduced Region Proposal Network (RPN), a fully convolutional network that shares full-image convolutional features with the detection network. It can be trained end-to-end to generate region proposals at nearly cost-free computations.

Unlike previous methods [7,9,10,11], which use pyramids of images or filters, Faster R-CNN [5] introduces the concept of anchor boxes. RPN adapts anchors (reference boxes) with three different scales and three different aspect ratios. The regression towards the output bounding boxes is achieved by comparing the proposed boxes with the anchors. RPN guides the Fast R-CNN network to the places where it is most likely to detect objects.

On the other hand, Lenc et al. [12] introduce one of the early attempts to accelerate the two-stage object detection networks. The study suggests dropping the region proposal section from the R-CNN [8] as it represents the bottleneck of the architecture. Instead of using a selective search for region proposals, the proposed system uses an image-independent list of candidate regions sampled from the distribution of the bounding boxes in the dataset. The investigation found that the CNN architecture by itself, without the fully connected layers, contains sufficient geometric information (spatial information) for accurate object detection. However, the accuracy of the proposed system without the region proposals network is negatively impacted.

Unlike detection approaches that modify classifier networks to perform detection, YOLO [13] (You Only Look Once) dealt with the object detection task as a regression problem in which bounding boxes are spatially separated and associated with class probabilities. YOLO approach uses a CNN to predict both the class probabilities and the bounding boxes from an image. It is a unified real-time object detection system with a design resembling GoogleNet [14] that needs one evaluation (forward pass) for predictions. Besides, the network can be trained end-to-end.

A disadvantage of the YOLO [13] approach is that it can only predict two bounding boxes for each grid cell. In addition, each grid cell can only have one class. As this spatial constraint, the detection of close objects is limited. Consequently, the network struggle with objects that appear in groups.

YOLO V2 [15], V3 [4] and V4 [16] are introduced to solve some of the challenges of YOLO V1 [13] and to enhance the detector performance. For instance, YOLO V2 uses Batch Normalisation (BN) [17], anchor boxes and multi-scale training. Whereas YOLO V3 uses residual connections [18] and Feature Pyramid Network (FPN) [19] with three predictions at different layers to process the image at different spatial resolutions. YOLO V4 [16] uses a different backbone network called CSPDarknet53. The introduced backbone network uses Cross Stage Partial Network (CSPNet) strategy to partition the feature map of the base layer into two parts and then merge them through a cross-stage hierarchy. The split and merge strategy allows for more gradient flow through the network. Comparisons of YOLO detector performances on different datasets are presented in [20,21,22]. RetinaNet [23] introduces two improvements over previous single-stage detectors. It uses Feature Pyramid Network (FPN) [19] and a novel focal loss instead of cross-entropy loss.

Small object detection represents a challenge for state-of-the-art detectors. These detectors are fine-tuned on datasets that contain large size objects. Besides, the base networks of these detectors are trained on general datasets such as ImageNet [24]. Studies show that state-of-the-art models [25,26,27,28,29], as well as standard datasets, such as PASCAL [30] and Microsoft COCO [31], do not give much consideration to small object detection. The performance of these models on small size objects is not deeply investigated as the evaluation of these models with the focus on the detection of small size objects is limited [27].

Moreover, the definition of small object size is not unified, which presents another challenge for researchers. Chen et al. [25] classify objects from the PASCAL dataset to be small if the ratio between the bounding box area to the image area, averaged over all the instances of that class, is in the range of 0.08% to 0.58%. This corresponds to 16 × 16 to 42 × 42 pixels. The small object can vary in size according to the image size, which is not constant for the PASCAL dataset. To compare, the median relative areas of the PASCAL dataset are between 1.38% to 46.0%.

Torralba et al. [32] introduce a dataset for tiny images with 32 × 32 pixels. Zhu et al. [26] follow the definition of the Microsoft COCO dataset for small-size objects to be equal to or less than 32 × 32 pixels. Microsoft COCO contains small objects, but they occupy large parts of the images. The variation in small-size objects definition is attributed to the dataset image size. For the PASCAL dataset, the image size varies. Whereas for the Microsoft COCO dataset, the image size is fixed and is equal to 640 × 480 pixels.

In the light of the previously mentioned definitions, this paper follows a new definition for small size objects. An object is categorised as small if its size equals to or is less than 42 × 42 pixels. This definition is adopted as the image size in the proposed object detection dataset is 512 × 512.

Chen et al. [25] is one of the first works that try to enhance the performance of R-CNN on small-size objects. The study introduces ContextNet, at which the region proposals and the context of the regions are forward propagated through two CNNs. Then the results of the two networks are concatenated. A limitation of the proposed system is that the two CNNs do not share any weights. Consequently, the system requires more training time and resources.

Several strategies have been introduced to enhance the detector performance on small-size objects, such as feature learning, context-based detection, data augmentation and training strategies. In addition, Generative-Adversarial Networks (GAN) [33] achieved good results on the task of small object detection. Tong et al. [34] review deep learning methods for small object detection. The review highlights the following remarks: multi-scale feature learning, context modelling and data augmentation can enhance the performance of state-of-the-art detection methods in the detection of small-size objects. Input image resolution and base networks have a great impact on detection performance. The combination of multiple techniques to enhance object detectors can further improve the performance [35,36]. Lastly, large datasets and the combination of multiple datasets can boost the detector to learn the better representation of small-size objects.

This paper investigates the performance of state-of-the-art object detection systems on the proposed dataset. The detailed investigation of different detector architectures and different training strategies gives a road map to choosing the most optimal system for a given application.

## 3. Methodology

### 3.1. System Architecture

Two of the most widely adopted object detection systems are used for investigation and performance comparison. The first detector is the one-stage YOLO V3 [4], while the second is the two-stage Faster R-CNN [5]. Both detectors use ResNet-18 as the base network.

The pipeline of the Faster R-CNN network consists of a feature extraction network, an RPN, and two sub-networks for class prediction and bounding box regression. The feature extraction network is a pretrained network that extracts the features from the input image. The RPN is trained to extract region proposals from the feature maps produced by the feature extraction network. Lastly, the classification and regression networks predict the class category and the bounding box of each region proposal. On the other hand, YOLO V3 uses a CNN to predict both the class probabilities and the bounding boxes from an image without the need for a region proposals step.

The choice of the feature extraction network is based on the application requirements. A deep network results in high accuracy but low processing speed and vice versa. Thus, the choice of the base network is a trade-off between accuracy and speed.

The choice of the feature extraction layer that feeds into the RPN is also a trade-off between the strength of the extracted features and the spatial resolution. High feature extraction layers (deep layers) down the network result in strong encoded features, but the object’s spatial information is lost. However, feature extraction layers up the network (early layers) have a better spatial resolution but weak encoded features. Empirical analysis can identify the optimal feature extraction layer for a specific application.

The detection of small size objects is a delicate task. The spatial information and features of small size objects are limited. Consequently, these objects can get lost as the feature maps are down-sampled through the network layers.

ResNet-18 [18] has been used as the feature extraction network in the experiments. ResNet-18 is the smallest version of the ResNet family. Nevertheless, it is a powerful network that uses residual blocks. It can achieve adequate processing speed with high accuracy. Residual blocks help to overcome deep network problems of vanishing and exploding gradients [37,38]. Residual blocks reuse the activations from previous layers until the adjacent layer learns its weights [18]. Four different feature extraction layers are used in the experiments to investigate the trade-off between spatial resolution and discriminative features: ‘res4a_relu’, ‘res4b_relu’, ‘res5a_relu’ and ‘res5b_relu’. These are the ReLU layers after the last four residual blocks of the ResNet-18 network.

### 3.2. Object Detection Dataset

The proposed object detection dataset contains 3292 images that are collected using a handheld camera. The object detection dataset is annotated at the bounding box level. Objects are categorised into eight classes. The number of object instances per class and the number of images which contain that object are shown in Table 1. The highest number of instances is for the ‘Door’ class. Whereas the lowest is for the ‘Push button’. Images of the dataset are shuffled and split randomly into 60% for training (1975), 10% for validation (330 images) and 30% for testing (987 images).

The proposed object detection dataset mainly contains small size objects. Objects with sizes less than or equal to 42 × 42 pixels are categorised as small-size objects. Objects bigger than 42 × 42 pixels and less than 96 × 96 pixels are categorised as medium size objects. Objects greater than 96 × 96 pixels are categorised as large-size objects. The definition of object sizes follows that of the Traffic [26] and COCO [31] datasets except for small size objects because the proposed images are larger than that of the traffic dataset. Consequently, the definition of small-size objects is 42 × 42 pixels instead of 32 × 32 pixels.

**Figure 1:**
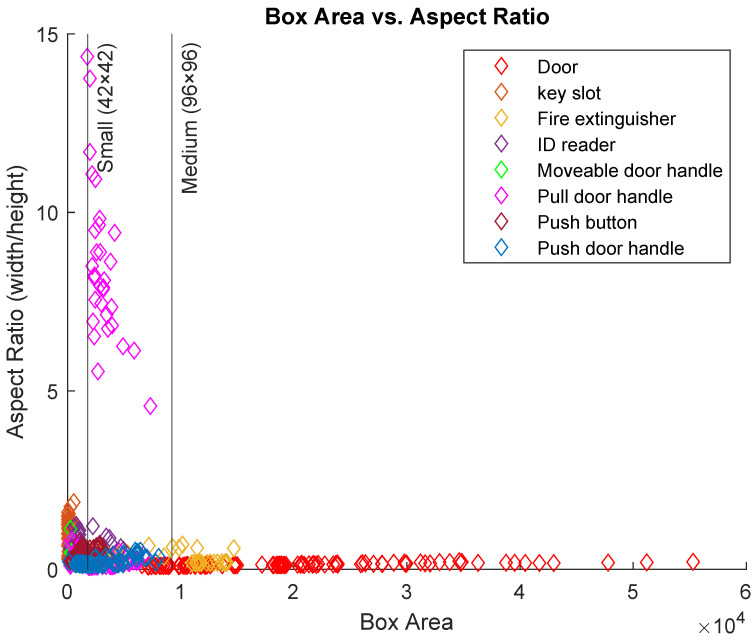
Sizes and aspect ratios of objects in the detection dataset.

Figure 1 shows the sizes and the aspect ratios of the object detection dataset. For ease of understanding and to better distinguish between objects, a set of randomly selected 100 objects from each category are displayed. The majority of the object sizes can be categorised as small and medium size objects. Consequently, the performance of state-of-the-art detection systems trained on large-size objects can differ due to the different nature of the proposed dataset. These detectors are designed with anchor boxes to accommodate general dataset objects (mainly large objects). Therefore, task-specific datasets need different designs for the anchor boxes and different techniques to capture small-size objects along with medium and large-size ones.

### 3.3. Training

#### 3.3.1. Training Parameters

Several training parameters are tried to find the optimal ones that can achieve the highest performance. The chosen training parameters for both detectors (Faster R-CNN and YOLO V3) are as follows: Stochastic Gradient Descent with Momentum (SGDM) is used as the training optimiser with 0.9 momentum. The Learning rate starts at 0.001 and then drops by a factor of 0.1 every six epochs. L2 regularisation of 0.005 is utilised to avoid overfitting. Training examples are shuffled every epoch to limit sequence memorising and avoid computing the gradients for the same batch of images.

#### 3.3.2. Data Augmentation

Data augmentation refers to increasing the number of images or instances of small size objects by image transformation that includes flipping, cropping, scaling, etc. The main idea is to extend the dataset with a large amount of data by increasing the representation of small size objects, which can help to boost the performance of detectors on small size objects [39].

Data augmentation techniques, such as image flipping can be employed to increase the variations and the number of training samples. Augmentation techniques can result in improved accuracy and enhanced model generalisation. Data augmentation techniques are applied to the training data only to produce a robust model and avoid evaluation bias. In the experiments, data augmentation techniques are employed on the training dataset by horizontal flipping of the images and associated boxes.

### 3.4. Evaluation

Average Precision (AP) that can be computed from the Precision (P) Recall (R) curves is the standard metric of evaluating object detectors. Precision can be calculated using Equation (1) as the ratio between True Positive (TP) instances to all positive instances. Whereas Recall can be calculated using Equation (2) as the ratio between TP instances to the sum of TP and False Negative (FN) instances (ground truth positives).

(1) $$P=\frac{TP}{TP+FP}$$

(2) $$R=\frac{TP}{TP+FN}$$

Intersection over Union is used to determine which detection is TP, False Positive (FP), or FN. If there is an overlap between the detected object bounding box and the ground truth bounding box above a certain threshold (in the experiments, the threshold is set to 0.5), the detection is considered a TP. If the IoU is less than the threshold, the detection is FP. Lastly, if there is an object, but it has not been detected, or the object is detected with a wrong category, then it is a FN.

AP is then calculated as the area under the Precision/Recall curve for a specific class of objects using Equation (3). A high AP value indicates the ability of the model to detect a specific class of objects efficiently and vice versa.

(3) $$AP=\int_{0}^{1}P\left(R\right)dR$$

Mean Average Precision (mAP) is used to assess the detector’s abilities over all the dataset objects. mAP can be calculated using Equation (4), where $AP_{k}$ is the AP for class *K* and *N* is the total number of classes. The metric reflects the detector’s performance over the whole dataset objects. In the experiments, AP for each class and mAP for all classes are reported.

(4) $$mAP=\frac{1}{N}\sum_{K=1}^{N}AP_{K}$$

## 4. Experimental Analysis and Discussion

Anchor boxes are a set of predefined boxes with different sizes and aspect ratios that represent the objects of the dataset. They are estimated from the training data and used as initial priors to enhance the predicted bounding boxes.

Anchor boxes are used to eliminate the need to scan the whole image using different sizes and aspect ratios sliding windows. Consequently, the whole image can be processed in a single propagation through the network, which enhances the overall prediction speed. Different sizes of anchor boxes enable the detection of multi-scale objects. The model predicts the offsets of the anchor boxes to refine the boxes’ locations and sizes.

The final detector output is produced by removing the anchor boxes that belong to the background. Moreover, other anchor boxes with confidence scores below a specific threshold are ignored. Lastly, the multiple detections of the same object are refined using the Non-Maximum Suppression (NMS) technique. Anchor boxes enable the prediction of multiple objects with different sizes and scales, besides overlapping objects.

Manually selecting the anchor boxes for the dataset is challenging as object groups are scattered with varying sizes and aspect ratios (Figure 1). A clustering algorithm, such as *k*-means [40], can group boxes of similar aspect ratios and sizes based on a specific metric. The Intersection over Union (IoU) distance metric is used to estimate the anchor boxes that better represent the dataset objects. IoU distance metric-based clustering algorithm can produce anchor boxes that fit the dataset objects efficiently as it is invariant to the boxes’ sizes [15]. Whereas other metrics such as Euclidean distance can lead to large errors when the boxes’ sizes increase [15].

**Figure 2:**
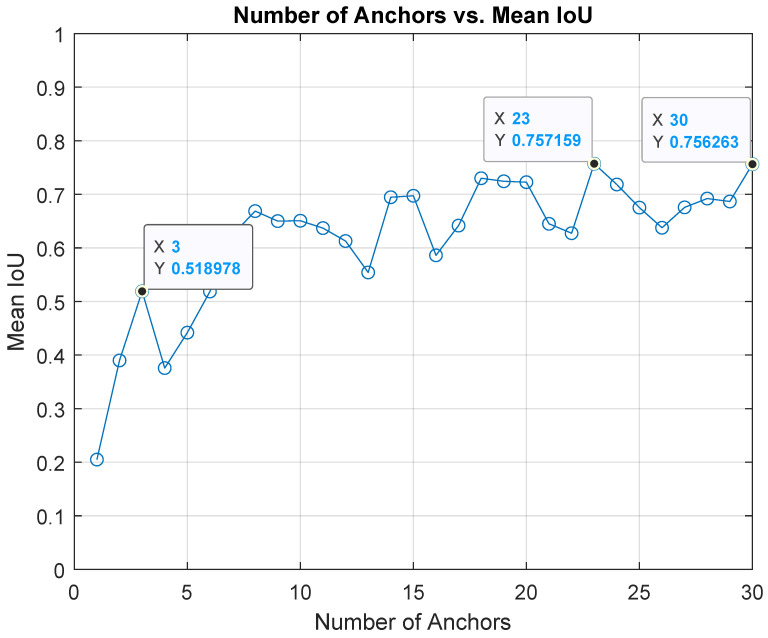
Estimated number of the anchor boxes with the corresponding mIoU.

The number of anchor boxes is a hyper-parameter that can be selected empirically. However, the mean IoU (mIoU) between the training data boxes and the estimated anchor boxes can be used to assess the number and validity of the estimated boxes. Figure 2 shows the estimated number of anchor boxes w.r.t the training data bounding boxes and the corresponding mIoU. The maximum number of anchor boxes is set to 30 as the mIoU plateaus or degrades after this point. Arbitrary increasing the number of anchor boxes can negatively affect the detector performance. Many anchor boxes can result in overfitting of the training data. Besides, the computation cost is directly proportional to the number of anchor boxes. Consequently, it is a trade-off process where the lowest number of anchor boxes that can achieve the highest mIoU is the objective.

A large number of anchor boxes results in low-performance detectors. Thus, a mIoU greater than 0.5 indicates adequate overlap between the training boxes and the estimated anchor boxes. Usually, marginal improvement can be achieved with many anchor boxes (mIoU starts to oscillate between 0.6 and 0.75 after 15 anchor boxes).

Three data points are selected to understand the impact of the anchor boxes on the detector performance (Figure 2). First, the point at which the mIoU is more than or equal to 0.5 with the lowest number of anchor boxes (number of anchor boxes = 3, mIoU = 0.518). Second, the point at which the highest mIoU can be achieved (number of anchor boxes = 23, mIoU = 0.757). Third, the point with the highest number of anchor boxes (number of anchor boxes = 30, mIoU = 0.756).

The adequate number of anchor boxes to achieve high accuracy, fast processing speed, or a trade-off between both metrics can be attained by analysing the dataset objects. Nevertheless, the application requirements are the main motive for choosing the number of anchor boxes.

Detectors are trained on a personal computer with an NVIDIA GeForce RTX 2080. Training time varies as the training process can be stopped early when the loss of the validation dataset plateaus or when the training process reaches the maximum number of epochs (30 epochs). The largest mini-batch size that can accommodate the available memory is sought. The largest mini-batch sizes are 2 and 16 in the case of the Faster R-CNN and YOLO V3 detectors, respectively. Table 2 and Table 3 show the training time of each model, the used mini-batch size, the stopping epoch, the trained model size and the number of layers.

Generally, Faster R-CNN detectors take significant training time compared to YOLO V3 detectors. The long training time is attributed to the detector architecture, which comprises an RPN attached to a Fast R-CNN [9]. This is translated into many layers and large footprints (Table 2). In contrast, the footprints and number of layers of YOLO V3 detectors vary depending on the feature extraction layer and the number of prediction heads. The smallest YOLO V3 detector has 48 layers and occupies a memory of 10 MB (Table 3).

The loss functions that have been used in the training process of Faster R-CNN and YOLO V3 are different, which can explain the difference in the results of Table 2 and Table 3. The objective function of Faster R-CNN follows the multi-task loss function of Fast R-CNN. However, it is minimised by a combination of the object classification loss and the bounding box regression loss (Equation (5)). The classification loss is a log loss over two classes, while the regression loss is the smooth L1 loss [9]. Smooth L1 loss is less sensitive to outliers compared to L2 loss, especially when regression targets are unbounded, which may cause exploding gradients when L2 loss is used.

(5) $$L_{\mathrm{Faster}\;R-\mathrm{CNN}\;}=L_{\mathrm{cls}\;}+L_{\mathrm{box}\;}$$

On the other hand, the YOLO V3 loss function optimises the training process over three different losses. Like Faster R-CNN, the classification loss is the binary cross-entropy loss. Unlike Faster R-CNN, Mean Square Error (MSE) is used for the bounding box loss. Besides, YOLO V3 introduces the bounding boxes objectness loss [4], which is an additional binary cross-entropy loss for the overlapping between the predicted and the ground truth boxes. Ideally, the objectness score should equal one when the best overlapping anchor box among all anchor boxes overlaps with the ground truth box. The predictions are ignored when other anchor boxes (not the best overlapping anchor box) overlap with the object box. This means that there is one anchor box assigned for each ground truth object [4].

(6) $$L_{\mathrm{YOLO}\;V3\;}=L_{\mathrm{cls}\;}+L_{\mathrm{box}\;}+L_{\mathrm{obj}\;}$$

Table 2 shows that the lowest validation loss achieved using Faster R-CNN is 0.192 using res5a_relu as the feature extraction layer with three anchor boxes. The lowest validation losses achieved using YOLO V3 with single, double and triple heads are 2.52, 2.35 and 2.38, respectively (Table 3). Like Faster R-CNN, the best-achieved validation loss YOLO V3 detectors use only three anchor boxes. Unlike Faster R-CNN, these YOLO detectors are trained using augmented data and with different feature extraction layers.

Earlier feature extraction layers in the network have higher spatial resolutions but may extract less semantic information compared to layers further down the network. High spatial resolution features are better for small and medium size objects but not for large size ones. In contrast, strong semantic information is important for large size objects. However, due to the successive down-sampling of the feature maps as the network goes deep, this information is lost for small objects. This makes the choice of the feature extraction layer a challenging task. As an example from Table 4 and Table 5, the AP of the smallest object in the proposed dataset (key slot) using earlier feature extraction layers such as res4a_relu or res4b_relu is significantly better than the AP when later layers such as res5a_relu or res5b_relu are used.

On the other hand, using res5a_relu or res5b_relu as the feature extraction layers on the largest size object in the proposed dataset (door) produces better AP than using res4a_relu or res4b_relu. This can be clearly seen from the YOLO V3 results (Table 5). In contrast, Faster R-CNN results do not reflect this fact (Table 4).

YOLO V3 detector can make predictions using multiple prediction heads over different scale feature maps in a similar approach to Feature Pyramid Network (FPN) [19]. The first head makes predictions over the first feature map. The second head makes predictions over a concatenation of the current feature map, after up-sampling and the previous feature map. The same approach is followed for the other heads. Thus, semantic information and fine-grained details can be obtained from the up-sampled and high-resolution feature maps. This approach allows the prediction of different scale objects, where small size objects can be detected from the high-resolution maps and large size objects can be extracted from strong semantic feature maps.

On the other hand, using a single feature map for prediction is less efficient than predictions over multiple feature maps, even with multiple scale anchor boxes (pyramid of anchors) that are used in Faster R-CNN [5]. Overall, the performance of YOLO V3 using single or multiple prediction heads are significantly better than that of Faster R-CNN for all object sizes.

Table 4 shows that the best Faster R-CNN detector achieved a mAP of 0.434. Whereas the best single-head YOLO V3 detector achieved a mAP of 0.765 (Table 5). A faster R-CNN detector uses res4b_relu as the feature extraction layer with 23 anchor boxes. Similarly, the YOLO V3 detector uses res4b_relu as the feature extraction layer but with only three anchor boxes. The best double and triple heads YOLO V3 detectors achieved a mAP of 0.786 and 0.818, respectively. Both of them use only three anchor boxes. Lastly, the overall best performance detector is YOLO V3 with triple heads and trained on augmented data.

Unlike the presented investigation, Liu et al. [41] survey shows that Faster R-CNN produces slightly better results than YOLO V3 on small object datasets [42,43,44]. Reflecting on the survey results [41] that use off-the-shelf Faster R-CNN and YOLO V3, the presented implementation unified the detectors set up in terms of using the same base networks, the same number of anchor boxes and the same feature extraction layers to facilitate the comparisons. Consequently, the detectors’ comparisons are robust and reliable. Moreover, the proposed dataset contains mainly small size objects but also medium and large size objects which can justify the differences.

On the other hand, the presented results align with the findings of Zeng et al. [2] on detecting tiny surface defects of the printed circuit board where the YOLO V3 detector can perform better on small-size objects. However, Faster R-CNN can better detect large-size objects [2]. Similarly, the proposed YOLO V3 detector can better detect small-size objects such as push buttons and key slots Table 4 and Table 5.

**Figure 3:**
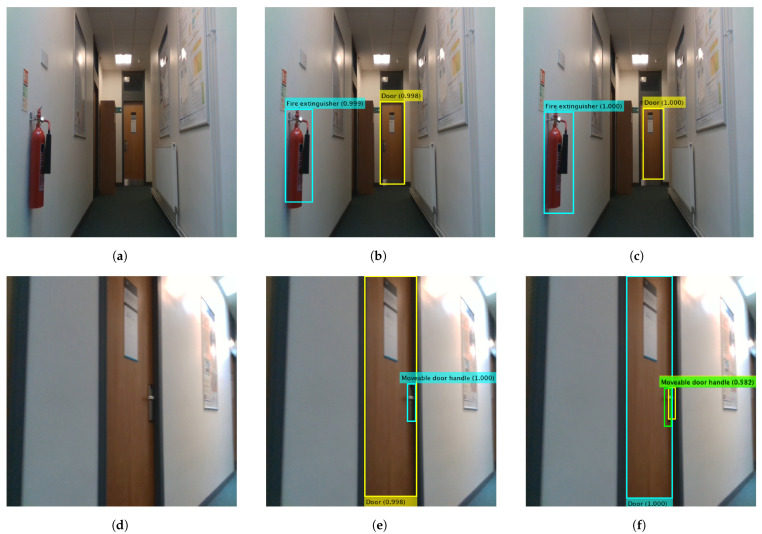
Prediction results of single head YOLO V3 and Faster R-CNN detectors on two test images. (**a**,**d**) Test images. (**b**,**e**) Output of YOLO V3. (**c**,**f**) Output of Faster R-CNN.

Figure 3 shows detection examples of the best performing Faster R-CNN (with 23 anchor boxes and res4b_relu feature extraction layer) and YOLO V3 (with three anchor boxes, res4b_relu feature extraction layer and trained using augmented training data) single head detectors on two test images. The two networks can predict the class categories and the bounding boxes with high confidence. However, Faster R-CNN predicts two bounding boxes for the same object (the moveable door handle in Figure 3f), one with a high confidence score of 0.99, while the second confidence score is relatively low (0.58). The confidence score threshold value used in the experiments is 0.5. A higher threshold value can discard the second box. Other Faster R-CNN detectors find it challenging to detect all the objects in the test images.

In contrast, other YOLO V3 detectors can localise all the objects in the test images with minor differences in the confidence scores. Another observation is that the predicted bounding box using Faster R-CNN for the fire extinguisher in Figure 3c covers the whole object, unlike the produced bounding box from the YOLO V3 detector (Figure 3b). In comparison, the bounding box for the door object in the same images is fully covered by the YOLO V3 detector and partially covered by the Faster R-CNN detector.

**Figure 4:**
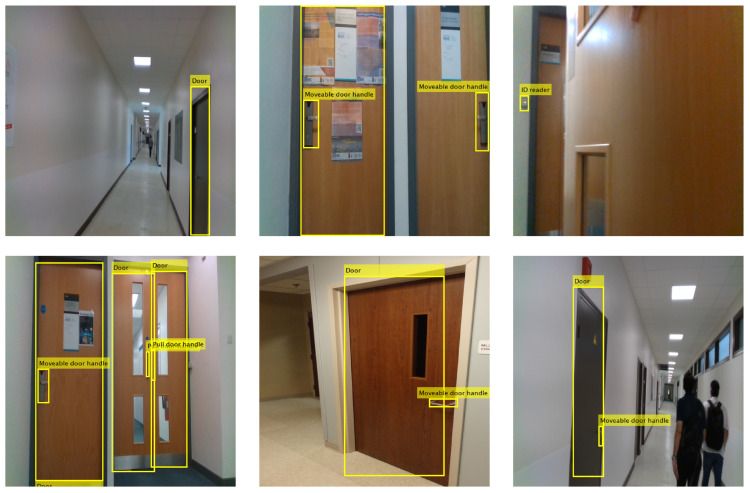
Prediction results of the best performing YOLO V3 detector with three heads.

Detection examples of the best performing three heads YOLO V3 detector are shown in Figure 4. The detector can localise small size objects, such as ID readers and pull door handles, along with large and medium size objects.

Detailed investigation of different detectors using different parameters can give insights into the suitable detector for a given application. YOLO V3 with three detection heads and three anchor boxes has achieved the best performance using data augmentation techniques during training on the proposed dataset. Moreover, the system needs less time for training compared to Faster R-CNN detectors.

It can be concluded from the results that small-size objects can be accurately detected using YOLO V3 detectors with an earlier feature extraction layer. On the other hand, increasing the number of anchor boxes is not enhancing the detector’s accuracy. However, feature extraction layers significantly impact the detector’s performance. Feature extraction layers greatly affect the detector’s ability to capture different size objects. An earlier layer can better localise small size objects, while a deeper layer can better encode large size objects. Consequently, multi-head detectors can capture different size objects efficiently because the predictions are made over several feature maps.

## 5. Visualisation of Detector Predictions

Visualisation techniques are essential tools to understand CNN behaviours. Reliable systems based on deep learning techniques need to reason about their predictions. For this reason, the transparency of the proposed systems is tested to ensure their robustness and accelerate their approval for real-life applications.

As an input pattern causes a given activation in the feature maps, Zeiler et al. [45] map this activation back to the input pixel space using deconvolutional networks [46]. The steps can be explained as follows: an input image is presented to the CNN and the features are computed through the networks’ layers. To analyse a given activation, all other activations in that layer are set to zero. Then the feature maps are passed to the attached deconvolutional layer. Finally, the input pixel space is reached through successive un-pooling, rectifying and filtering operations to reconstruct the layer’s activity.

Gradients approach [47], also known as backpropagation or saliency method, visualises the derivatives of the target object score with respect to the input image (Equation (7)). Saliency maps are generated for the trained network and not during the training process (i.e., the networks’ weights are constant). Backpropagation is the process of increasing or decreasing networks’ weights to minimise the loss of function during the training process [48]. Saliency maps return the spatial discriminative pixels locations of a particular class in an image.

Although Gradient heatmaps are computationally faster than Occlusion [46] as it only needs one backward propagation through the network, they do not fully explain the image prediction. The calculated map measures pixels change that would make an image belong to a specific category. However, it does not explain the classifier decision as argued by [49] or the direct relation to the variation of the output [50,51].

DeconvNet approach [46], which zeros negative values of the top gradients and backpropagation [47], which zeros negative values from the bottom inputs, are then combined to produce Guided Backpropagation (GBP) [52], which zeros both negative values (Equation (8)). The signal from higher layers guides the backpropagation; hence the name is derived. It works as the switches of the DeconvNet approach [46]. Doing so prevents negative gradients from flowing back, which can undesirably impact the activation’s visualisation.

Many approaches based on Gradients are proposed, such as Integrated Gradients (IG) [53]. IG [53] approach accumulates gradients over scaled versions of the input that follow a baseline defined by the user, i.e., they integrate the gradients of all points that fall on the straight-line path from the baseline to the input (Equation (9)).

(7) $$Z_{ij}^{Grad}:=\frac{\partial Y^{c}\left(X\right)}{\partial X_{ij}}$$

(8) $$Z_{ij}^{GBP}:=\left\{\begin{array}{cc} \frac{\partial Y^{c}\left(X\right)}{\partial X_{ij}} & \forall \left(X_{ij}>0\;\&\;\frac{\partial Y^{c}}{\partial X_{ij}}>0\right)\\ 0 & \;\mathrm{otherwise}\; \end{array}\right\}$$

(9) $$Z_{ij}^{IG}:=\left(X_{ij}-{\bar{X}}_{ij}\right)\int_{\alpha =0}^{1}\frac{\partial Y^{c}\left({\bar{X}}_{ij}+\alpha \left(X_{ij}-{\bar{X}}_{ij}\right)\right)}{\partial X_{ij}}d\alpha$$

where,

*X*: Input image
$X_{ij}$: Image pixel
${\bar{X}}_{ij}$: Baseline image pixel
$Y^{c}$: Output prediction for class c
α: A real value parameter that defines the path steps between the baseline image and the input image
$Z_{ij}$: Generated heatmap
Gradient calculations following the chain-rule.

Gradient-based methods, such as saliency [47], GBP [52] and IG [53], can produce high-resolution heatmaps. However, each approach captures specific features that contribute to the overall output. Two techniques are proposed to attain the benefits of these methods: weighted sum of gradient approach (WS-Grad) and concatenation of gradient approach (Concat-Grad).

### 5.1. Weighted Sum of Gradients Approach (WS-Grad)

**Figure 5:**
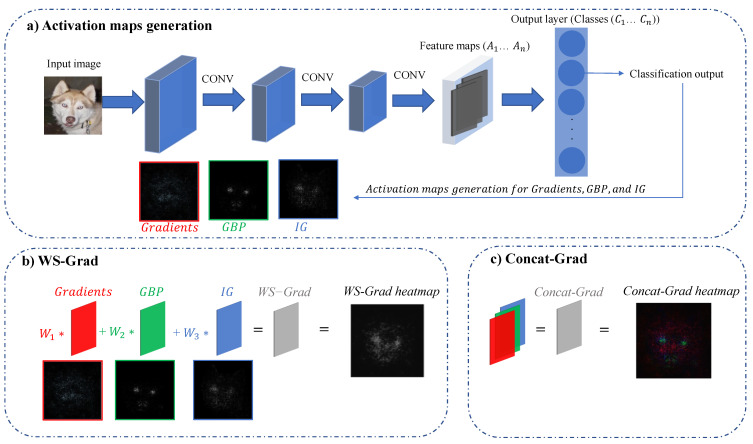
Proposed visualisation approaches using an image from a classification dataset. (**a**) activation maps generation, (**b**) Weighted sum of gradients approach (WS-Grad) and (**c**) Concatenation of gradients approach (Concat-Grad).

Figure 5b shows the weighted sum gradients approach. First, gradients-based heatmaps are generated (Figure 5a). Then each map is scaled by a weight that the user can determine to highlight specific features because different maps can highlight various elements (Equation (10)). For example, saliency maps (Gradient approach) highlight all the features that contribute equally to the prediction. However, GBP focuses on the most discriminative features and ignores supplementary ones. IG approach accumulates gradients over scaled versions of the input that follow a baseline defined by the user. The flexibility of choosing the weights is a powerful tool that can be utilised differently according to the application. The produced heatmap is more expressive than the individual ones, where the most important features are strongly highlighted with high resolution.

(10) $$Z_{ij}^{WS-Grad}=W_{1}*Z_{ij}^{\mathrm{Grad}}+W_{2}*Z_{ij}^{\mathrm{GBP}}+W_{3}*Z_{ij}^{IG}$$

### 5.2. Concatenation of Gradients Approach (Concat-Grad)

Figure 5c shows the concatenated gradients approach. Similar to WS-Grad, Gradient-based heatmaps are generated. Then, the generated maps are weighted and concatenated as a single image with three channels (Equation (11)).

The Concat-Grad approach produces high-resolution heatmaps where all the important features using different visualisation approaches can be seen and identified in one image. The generated map has three channels (similar to an RGB image). Consequently, the produced heatmap reflects each map using a different colour. This means Gradient, GBP and IG features are depicted in red, green and blue colours, respectively. The novelty of this method can be seen in the ability to distinguish various features of different approaches using distinctive colours in one map, which is very informative as it gives better insights into the important features and their corresponding approach.

(11) $$Z_{ij}^{Concat-Grad}={\left[\begin{array}{c} W_{1}*Z_{ij}^{Grad}\\ W_{2}*Z_{ij}^{GBP}\\ W_{3}*Z_{ij}^{IG} \end{array}\right]}^{⊤}$$

### 5.3. Visualisation Results

To validate the reliability of the best single-head YOLO V3 detector, novel techniques for visualising the network decisions are applied. The proposed techniques can be applied to classification tasks like other visualisation techniques [54]. However, applying them to different tasks, such as object detection, is a novel contribution.

The output of a YOLO V3 single head detector is N×N×[3×(4+1+8)] where *N* represents the convolution filter size, 3 represents the number of anchor boxes, 4 represents the bounding box offsets, 1 represents the objectness score and 8 represents the scores of classes. The convolutional filter size is 16, resulting in an output tensor of dimension 16×16×39. The target object score needs to be tracked back through the network layers to find the contributing input pixels to the target object score. However, the final output scores are obtained by multiplying the confidence score and the objectness score.

Tracking the final output scores through the output tensor is challenging because of the post-processing step that extracts the confidence and objectness score from the output tensor and multiplies them. This post-processing step needs to be reversed. Besides, the two values that produce the final output score need to be tracked back (using backpropagation) through the network to find the corresponding contributing features. In contrast, the final output score can be directly tracked in classification tasks that use the softmax layer in the output, whereas YOLO V3 does not have any softmax layers. The locations of the confidence and objectness scores are identified by analysing the output tensor. Consequently, the output target score has become feasible to be tracked through the detector network. In other words, by identifying the location of the confidence and objectness score in the output tensor, the output score of the target object has become trackable.

**Figure 6:**
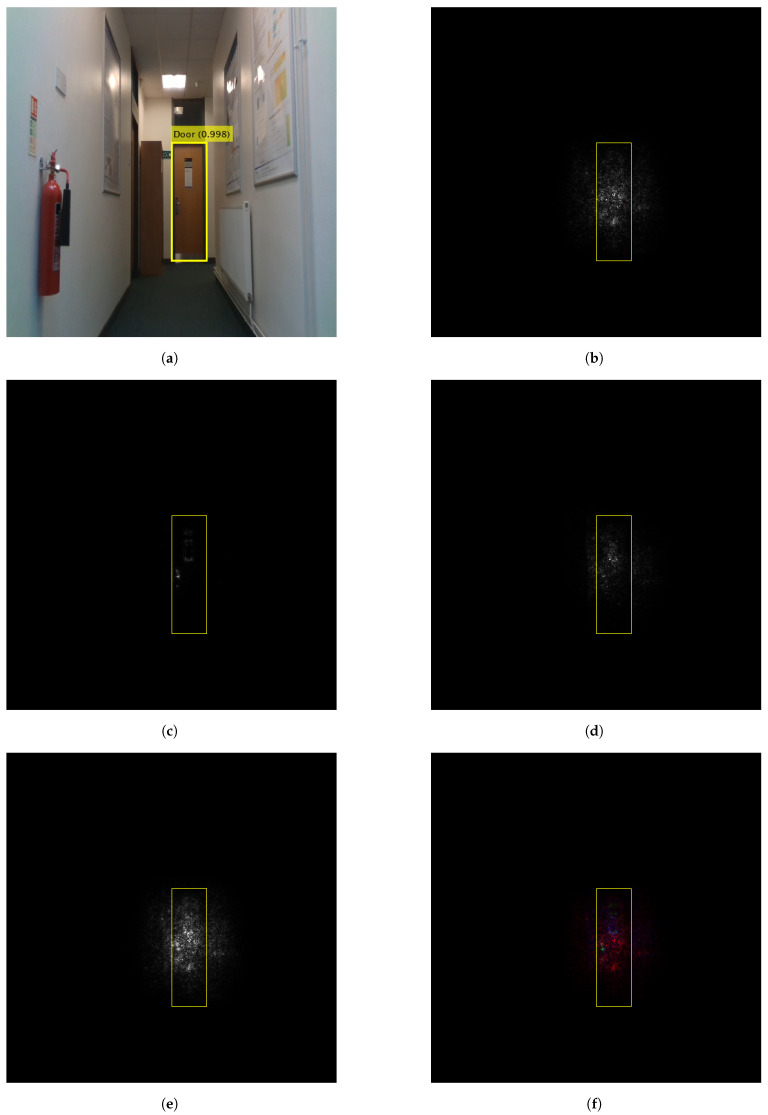
Gradients based attribution methods and the proposed methods to visualise the door prediction using YOLO V3. (**a**) YOLO prediction. (**b**) Gradient. (**c**) GBP. (**d**) IG. (**e**) WS-Grad (proposed). (**f**) Concat-Grad (proposed).

**Figure 7:**
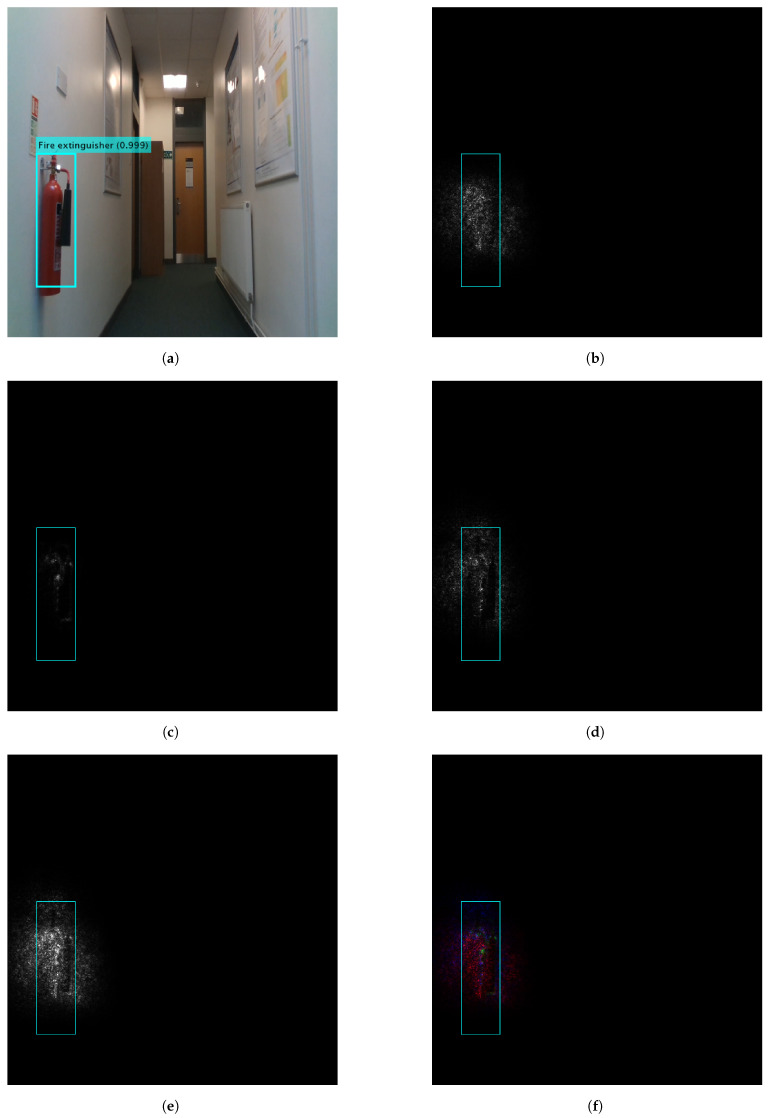
Gradients based attribution methods and the proposed methods to visualise the fire extinguisher prediction using YOLO V3. (**a**) YOLO prediction. (**b**) Gradient. (**c**) GBP. (**d**) IG. (**e**) WS-Grad (proposed). (**f**) Concat-Grad (proposed).

Figure 6 and Figure 7 show the contributing pixels to the detector predictions of the door and the fire extinguisher, respectively. The weighting parameter of the proposed methods is set to one for a fair comparison with state-of-the-art methods. Gradient [47], GBP [52], IG [53], WS-Grad and Concat-Grad attribution maps are compared. Gradient, GBP and IG show specific individual features. However, the proposed methods show comprehensive maps that contain all of the contributing pixels. Furthermore, the Concat-Grad method shows the features of each individual method in different colours on a single map which enriches the output and makes it more descriptive and understandable.

As an example, the WS-Grad heatmap of the door in Figure 6e highlights the important common features among all other individual visualisation techniques. On the other hand, the Concat-Grad technique visualises the individual features in different colours. This allows the identification of correct features that contributes to the network decision. For instant, the Gradient method that is depicted in red shows that the network-based its decision on the context information. Whereas the GBP features that are depicted in green show that the network-based its decision on the door handle. Lastly, the IG, which is depicted in blue, shows that the network-based its decision on the door panel. While some features should not contribute to the network decision, such as the door panel, visualising different contributing features using the proposed method can greatly help to understand and enhance the detector performance. In this case, the detector performance and ability to generalise can be enhanced further by training on door images without panels.

Generally, the research area of visualising the network decision for object detection tasks has not been adequately explored. The proposed techniques can help researchers in the field of object detection to understand and trust the decisions of their systems. Besides, visualising the detector prediction can help to debug the system in the case of bias or error.

One limitation of this study is in the visualisation of Faster R-CNN predictions. Faster R-CNN detectors contain some layers that are challenging to reverse (backpropagate the output through them) such as region proposal and region of interest pooling layers. Further, the 4-step alternating training method [5] that is used by Faster R-CNN to train the region proposal and region classification sub-networks separately makes it challenging to track the activations through the network layers.

## 6. Conclusions

This paper presents a comprehensive investigation of object detector performance. Detector performance using different feature extraction layers and a different number of anchor boxes is investigated. Besides, the impact of training the detectors using data augmentation techniques is highlighted.

Data augmentation positively impacts the generated detectors and results in lower validation loss compared to detectors trained on data without augmentation techniques. However, increasing the number of anchor boxes does not enhance the detector’s performance. In contrast, it can negatively impact performance. YOLO V3 detectors with multi-prediction heads achieved the best performance. Furthermore, YOLO V3 detectors have fewer layers, less footprint and train faster than Faster R-CNN detectors.

Feature extraction layer can significantly impact the ability of the detector to localise different size objects. An earlier feature extraction layer can better detect small size objects, as it can preserve spatial information. Whereas later layers are better with large size objects because it better encodes the object’s features. Successive down-sampling of features as the object propagates through the network layers strengthen the encoded features, but spatial information of small size object can be lost. Consequently, earlier feature extraction layer is advisable with small-size object detection applications.

The paper greatly contributes to the visualisation and explanation techniques applied to the object detection task as research in this area is very limited. It is important to attain not only an accurate system but also a system that can explain its predictions. Black box systems, like deep convolutional networks, must provide adequate insights into the system’s predictions. Developers, policymakers and legislators often require a certain level of system transparency to approve/appreciate such technologies and can self-assuredly conclude that the underlying system is robust and reliable. Consequently, these kinds of transparent systems can be approved and used for critical real-life applications. The proposed explanation techniques help achieve this by providing high-resolution and sharp heatmaps for the contributing features to the network decision compared to the state-of-the-art ones. This can greatly help to understand and explain the detector’s behaviour.

## Figures and Tables

**Table 1:** Number of instances per class and the image count.

Class	No# of Instance	No# of Images
Door	3443	2548
Pull door handle	362	227
Push button	92	92
Moveable door handle	2035	1797
Push door handle	437	367
Fire extinguisher	536	536
Key slot	826	763
ID reader	500	485

**Table 2:** Training details (Faster R-CNN with ResNet-18 using mini-batch size of 2).

Model	Feature Layer	No# of Anchors	Training Time (≈Hours)	Stopping Epoch	Size (MB)/ No# of Layers	Validation Loss
Faster R-CNN	res4a_relu	30	16.3	15	42/82	0.681
Faster R-CNN *	res4a_relu	30	18.6	15	42/82	0.719
Faster R-CNN	res4b_relu	30	14	17	42/82	0.575
Faster R-CNN *	res4b_relu	30	17.5	18	42/82	0.627
Faster R-CNN	res5a_relu	30	5	15	48.5/82	0.369
Faster R-CNN *	res5a_relu	30	4	11	48.5/82	0.399
Faster R-CNN	res5b_relu	30	2.5	14	48.5/82	0.396
Faster R-CNN *	res5b_relu	30	3	17	48.5/82	0.479
Faster R-CNN	res4a_relu	23	34.7	9	42/82	0.639
Faster R-CNN *	res4a_relu	23	56	14	42/82	0.639
Faster R-CNN	res4b_relu	23	41.5	19	42/82	0.521
Faster R-CNN *	res4b_relu	23	32	15	42/82	0.529
Faster R-CNN	res5a_relu	23	4.5	13	48.4/82	0.303
Faster R-CNN *	res5a_relu	23	5	13	48.4/82	0.311
Faster R-CNN	res5b_relu	23	2.7	15	48.4/82	0.318
Faster R-CNN *	res5b_relu	23	2.3	14	48.4/82	0.342
Faster R-CNN	res4a_relu	3	17.6	10	41.9/82	0.366
Faster R-CNN *	res4a_relu	3	23.3	14	41.9/82	0.380
Faster R-CNN	res4b_relu	3	12	10	41.9/82	0.310
Faster R-CNN *	res4b_relu	3	11.1	11	41.9/82	0.328
Faster R-CNN	res5a_relu	3	1	9	48.2/82	**0.192**
Faster R-CNN *	res5a_relu	3	1.1	12	48.2/82	0.201
Faster R-CNN	res5b_relu	3	0.5	6	48.2/82	0.336
Faster R-CNN *	res5b_relu	3	0.4	6	48.2/82	0.264

* System trained on augmented data. MB = Megabyte.

**Table 3:** Training details (YOLO V3 with ResNet-18 using mini-batch size of 16).

Model	Feature Layer	No# of Anchors	Training Time (≈Hours)	Stopping Epoch	Size (MB)/ No# of Layers	Validation Loss
YOLO V3	res4a_relu	30	0.9	25	10.6/48	7.91
YOLO V3 *	res4a_relu	30	1.1	29	10.6/48	6.40
YOLO V3	res4b_relu	30	1	24	14.8/55	6.89
YOLO V3 *	res4b_relu	30	1.1	28	14.8/55	5.23
YOLO V3	res5a_relu	30	1.1	25	41.1/64	4.68
YOLO V3 *	res5a_relu	30	1.2	28	41.1/64	3.93
YOLO V3	res5b_relu	30	1.3	27	57.9/71	4.20
YOLO V3 *	res5b_relu	30	1.5	30	57.9/71	3.57
YOLO V3	res4a_relu	23	0.8	24	10.4/48	7.64
YOLO V3 *	res4a_relu	23	1.1	28	10.4/48	6.25
YOLO V3	res4b_relu	23	0.9	23	14.6/55	6.31
YOLO V3 *	res4b_relu	23	1.1	27	14.6/55	5.17
YOLO V3	res5a_relu	23	1.2	27	40.8/64	4.37
YOLO V3 *	res5a_relu	23	1.3	30	40.8/64	3.90
YOLO V3	res5b_relu	23	1.4	29	57.6/71	3.96
YOLO V3 *	res5b_relu	23	1.5	30	57.6/71	3.40
YOLO V3	res4a_relu	3	0.8	24	10/48	6.22
YOLO V3 *	res4a_relu	3	0.9	27	10/48	4.73
YOLO V3	res4b_relu	3	0.9	26	14.2/55	4.67
YOLO V3 *	res4b_relu	3	0.9	25	14.2/55	3.75
YOLO V3	res5a_relu	3	1.3	30	39.8/64	3.27
YOLO V3 *	res5a_relu	3	1.2	28	39.8/64	2.85
YOLO V3	res5b_relu	3	1.2	25	56.6/71	2.74
YOLO V3 *	res5b_relu	3	1.5	30	56.6/71	**2.52**
YOLO V3	res4a&5a_relu	3	1.2	24	43.2/73	3.56
YOLO V3 *	res4a&5a_relu	3	1.2	24	43.2/73	2.95
YOLO V3	res4a&5b_relu	3	1.5	27	60/80	2.95
YOLO V3 *	res4a&5b_relu	3	1.5	28	60/80	**2.35**
YOLO V3	res4b&5a_relu	3	1.3	27	43.2/73	3.52
YOLO V3 *	res4b&5a_relu	3	1.5	28	43.2/73	2.97
YOLO V3	res4b&5b_relu	3	1.5	27	60/80	2.93
YOLO V3 *	res4b&5b_relu	3	1.6	27	60/80	2.51
YOLO V3	res4a&4b&5a_relu	3	1.4	16	46.4/82	3.79
YOLO V3 *	res4a&4b&5a_relu	3	1.8	30	46.4/82	**2.38**
YOLO V3	res4a&4b&5b_relu	3	1.4	23	63.2/89	3.28
YOLO V3 *	res4a&4b&5b_relu	3	1.7	28	63.2/89	2.94
YOLO V3	res4a&5a&5b_relu	3	1.5	24	72.9/89	3.27
YOLO V3 *	res4a&5a&5b_relu	3	1.7	27	72.9/89	2.46
YOLO V3	res4b&5a&5b_relu	3	1.9	30	72.9/89	3.32
YOLO V3 *	res4b&5a&5b_relu	3	1.9	30	72.9/89	2.57

* System trained on augmented data. MB = Megabyte.

**Table 4:** Detectors detailed results on the test set (Faster R-CNN with ResNet-18).

Model	Feature Layer	No# of Anchors	*AP* for Each Class								*mAP*
			Door	Key Slot	Fire Extinguisher	ID Reader	Moveable Door Handle	Pull Door Handle	Push Button	Push Door Handle	
Faster R-CNN	res4a_relu	30	0.911	0.061	0.587	0.315	0.330	0.354	0.000	0.205	0.345
Faster R-CNN *	res4a_relu	30	0.916	0.066	0.639	0.285	0.379	0.340	0.000	0.250	0.359
Faster R-CNN	res4b_relu	30	0.911	0.089	0.666	0.274	0.404	**0.415**	0.000	**0.289**	0.381
Faster R-CNN *	res4b_relu	30	0.905	0.088	0.709	0.215	0.394	0.379	0.000	0.238	0.366
Faster R-CNN	res5a_relu	30	0.810	0.000	0.407	0.071	0.081	0.000	0.000	0.029	0.174
Faster R-CNN *	res5a_relu	30	0.799	0.000	0.396	0.088	0.056	0.000	0.000	0.000	0.167
Faster R-CNN	res5b_relu	30	0.504	0.000	0.154	0.000	0.011	0.000	0.000	0.000	0.083
Faster R-CNN *	res5b_relu	30	0.432	0.000	0.097	0.000	0.002	0.000	0.000	0.000	0.066
Faster R-CNN	res4a_relu	23	0.883	0.092	0.673	0.190	0.298	0.317	0.000	0.280	0.341
Faster R-CNN *	res4a_relu	23	0.890	0.086	0.633	0.203	0.411	0.351	0.000	0.244	0.352
Faster R-CNN	res4b_relu	23	**0.924**	0.068	0.742	0.426	**0.427**	0.384	**0.279**	0.222	**0.434**
Faster R-CNN *	res4b_relu	23	0.910	**0.109**	0.642	0.285	0.409	0.344	0.197	0.242	0.392
Faster R-CNN	res5a_relu	23	0.780	0.000	0.388	0.132	0.069	0.000	0.000	0.071	0.180
Faster R-CNN *	res5a_relu	23	0.777	0.000	0.361	0.000	0.041	0.000	0.000	0.000	0.147
Faster R-CNN	res5b_relu	23	0.505	0.000	0.242	0.068	0.035	0.000	0.000	0.000	0.106
Faster R-CNN *	res5b_relu	23	0.431	0.000	0.088	0.000	0.009	0.000	0.000	0.000	0.066
Faster R-CNN	res4a_relu	3	0.873	0.018	0.692	0.211	0.390	0.202	0.000	0.082	0.308
Faster R-CNN *	res4a_relu	3	0.869	0.066	0.729	0.177	0.361	0.356	0.000	0.108	0.333
Faster R-CNN	res4b_relu	3	0.909	0.105	0.602	**0.481**	0.396	0.338	0.000	0.190	0.378
Faster R-CNN *	res4b_relu	3	0.889	0.034	**0.754**	0.180	0.418	0.354	0.000	0.233	0.357
Faster R-CNN	res5a_relu	3	0.745	0.000	0.415	0.000	0.028	0.000	0.000	0.000	0.148
Faster R-CNN *	res5a_relu	3	0.749	0.000	0.257	0.000	0.033	0.000	0.000	0.000	0.130
Faster R-CNN	res5b_relu	3	0.123	0.000	0.116	0.000	0.004	0.000	0.000	0.000	0.030
Faster R-CNN *	res5b_relu	3	0.502	0.000	0.127	0.000	0.002	0.000	0.000	0.000	0.079

* System trained on augmented data.

**Table 5:** Detectors detailed results on the test set (YOLO V3 with ResNet-18).

Model	Feature Layer	No# of Anchors	*AP* for Each Class								*mAP*
			Door	Key Slot	Fire Extinguisher	ID Reader	Moveable Door Handle	Pull Door Handle	Push Button	Push Door Handle	
YOLO V3	res4a_relu	30	0.516	0.462	0.714	0.657	0.632	0.536	0.500	0.458	0.559
YOLO V3 *	res4a_relu	30	0.561	0.556	0.787	0.739	0.744	0.543	0.566	0.470	0.620
YOLO V3	res4b_relu	30	0.573	0.510	0.788	0.751	0.716	0.566	0.666	0.538	0.638
YOLO V3 *	res4b_relu	30	0.670	0.546	0.797	0.807	0.779	0.575	0.689	0.648	0.668
YOLO V3	res5a_relu	30	0.721	0.156	0.843	0.618	0.593	0.519	0.833	0.437	0.590
YOLO V3 *	res5a_relu	30	0.786	0.230	0.838	0.670	0.616	0.510	0.739	0.412	0.600
YOLO V3	res5b_relu	30	0.760	0.192	0.856	0.604	0.621	0.482	0.797	0.437	0.593
YOLO V3 *	res5b_relu	30	0.809	0.196	0.826	0.634	0.617	0.462	0.774	0.400	0.589
YOLO V3	res4a_relu	23	0.563	0.424	0.720	0.687	0.633	0.578	0.566	0.407	0.572
YOLO V3 *	res4a_relu	23	0.609	0.474	0.754	0.680	0.712	0.539	0.493	0.505	0.596
YOLO V3	res4b_relu	23	0.632	0.445	0.766	0.731	0.675	0.658	0.600	0.455	0.620
YOLO V3 *	res4b_relu	23	0.699	0.496	0.825	0.760	0.743	0.567	0.622	0.515	0.653
YOLO V3	res5a_relu	23	0.771	0.194	0.806	0.623	0.622	0.517	0.740	0.405	0.584
YOLO V3 *	res5a_relu	23	0.791	0.176	0.753	0.645	0.580	0.454	0.800	0.405	0.575
YOLO V3	res5b_relu	23	0.809	0.192	0.826	0.617	0.592	0.477	0.833	0.527	0.609
YOLO V3 *	res5b_relu	23	0.821	0.166	0.777	0.654	0.591	0.471	0.866	0.362	0.588
YOLO V3	res4a_relu	3	0.652	0.502	0.805	0.762	0.721	0.643	0.758	0.555	0.674
YOLO V3 *	res4a_relu	3	0.726	0.523	0.882	0.817	0.767	0.572	0.849	0.673	0.726
YOLO V3	res4b_relu	3	0.745	0.558	0.846	0.804	0.750	0.641	0.755	0.686	0.723
YOLO V3 *	res4b_relu	3	0.815	0.582	**0.922**	0.868	0.777	0.665	0.755	0.736	**0.765**
YOLO V3	res5a_relu	3	0.834	0.195	0.859	0.712	0.692	0.562	0.861	0.511	0.653
YOLO V3 *	res5a_relu	3	0.855	0.188	0.877	0.725	0.728	0.501	0.691	0.483	0.631
YOLO V3	res5b_relu	3	0.873	0.212	0.874	0.766	0.736	0.523	0.695	0.581	0.657
YOLO V3 *	res5b_relu	3	0.887	0.189	0.881	0.768	0.732	0.528	0.833	0.522	0.667
YOLO V3	res4a&5a_relu	3	0.834	0.499	0.914	0.792	0.754	0.599	0.800	0.512	0.713
YOLO V3 *	res4a&5a_relu	3	0.856	0.595	0.911	0.843	0.805	0.517	0.866	0.682	0.759
YOLO V3	res4a&5b_relu	3	0.879	0.636	0.915	0.856	0.821	0.617	0.769	0.688	0.772
YOLO V3 *	res4a&5b_relu	3	0.902	0.614	0.908	0.865	0.843	0.630	0.777	0.743	0.785
YOLO V3	res4b&5a_relu	3	0.835	0.537	0.904	0.825	0.767	0.613	0.798	0.574	0.731
YOLO V3 *	res4b&5a_relu	3	0.862	0.582	0.914	**0.904**	0.812	0.606	0.850	0.682	0.776
YOLO V3	res4b&5b_relu	3	0.888	0.573	**0.922**	0.855	0.807	0.684	0.881	0.679	**0.786**
YOLO V3*	res4b&5b_relu	3	0.895	0.604	0.915	0.892	0.844	0.621	0.753	0.707	0.778
YOLO V3	res4a&4b &5a_relu	3	0.847	0.510	0.896	0.803	0.766	0.695	0.793	0.667	0.747
YOLO V3 *	res4a&4b &5a_relu	3	0.907	**0.673**	0.937	0.862	**0.869**	0.638	**0.893**	**0.766**	**0.818**
YOLO V3	res4a&4b &5b_relu	3	0.892	0.454	0.896	0.773	0.759	**0.724**	0.757	0.607	0.732
YOLO V3 *	res4a&4b &5b_relu	3	**0.917**	0.571	0.941	0.887	0.831	0.655	0.775	0.730	0.788
YOLO V3	res4a&5a &5b_relu	3	0.878	0.593	0.921	0.792	0.775	0.616	0.775	0.651	0.750
YOLO V3 *	res4a&5a &5b_relu	3	0.892	0.561	**0.954**	0.877	0.833	0.612	0.749	0.641	0.764
YOLO V3	res4b&5a &5b_relu	3	0.877	0.539	0.902	0.787	0.770	0.552	0.782	0.535	0.718
YOLO V3 *	res4b&5a &5b_relu	3	0.900	0.516	0.932	0.847	0.837	0.601	0.870	0.709	0.776

* System trained on augmented data.
